# Improving the Timeliness and Quality of Electrocardiogram Acquisition, Interpretation, and Documentation in the Emergency Department: A Closed-Loop Quality Improvement Study at Hasahesa Teaching Hospital, Sudan

**DOI:** 10.7759/cureus.108917

**Published:** 2026-05-15

**Authors:** Amro Osama Fathi Mohammed, Fatima Dahab, Elaf Abdelrahman, Obada Alwaleed Ahmed Mohamed, Zeinab Osman, Khattab Mohammed Aboudi Ahamed, Yahya Abozamr, Omnia Hassan, Hala Osman, Dalia Mohamed, Mihad Babiker, Mohja Fadulamoula, Wafa Abdelkarim, Amna Fadlemula, Sahar Abdulrahman, Doaa Suliman, Doaa Mohamed, Malath Elamin, Doaa Khalid, Thowaiba Saidahmed, Arwa Alzubair, Thekra Mohamed

**Affiliations:** 1 Internal Medicine, Al Neelain University, Khartoum, SDN; 2 Emergency Medicine, Hasahesa Teaching Hospital, Al-Hasaheisa, SDN; 3 General Medicine, Al Neelain University, Khartoum, SDN; 4 Internal Medicine, Hasahesa Teaching Hospital, Al-Hasaheisa, SDN; 5 Medical Education, Al Neelain University, Khartoum, SDN

**Keywords:** acute coronary syndrome, electrocardiogram, emergency department, hasahesa teaching hospital, quality improvement

## Abstract

Background

Timely acquisition and accurate interpretation of electrocardiograms (ECGs) are essential for the early diagnosis and management of acute coronary syndromes (ACS) in emergency departments (EDs). Delays in ECG performance and deficiencies in documentation can compromise patient outcomes and quality of care, particularly in resource-limited settings.

Objective

This study aims to evaluate and improve the timeliness of ECG acquisition, interpretation, and documentation in the ED through a structured quality improvement approach.

Methods

A closed-loop quality improvement project was conducted at Hasahesa Teaching Hospital, Sudan, using a two-cycle clinical audit design structured around the Plan-Do-Study-Act (PDSA) framework. Data were collected prospectively over two audit cycles using a total population sampling approach. The first cycle was conducted over three months from June to August 2025 and included 90 patients. Following a two-month intervention phase, a second cycle was conducted over three months and included 105 patients, yielding a total of 195 cases. Key parameters assessed included time from arrival to ECG acquisition, time to interpretation and documentation, clinical indication, and completeness of ECG documentation. Targeted interventions included staff education, workflow optimization, and reinforcement of standardized documentation practices.

Results

The proportion of patients receiving ECG within 10 minutes of arrival increased significantly from 43/90 (47.8%) in the first cycle to 90/105 (85.7%) in the second cycle (p<0.001). The mean time from arrival to ECG acquisition decreased from 25.4 minutes to 4.5 minutes. Significant improvements were observed across all documentation parameters, including ST-segment documentation (25/90 (27.8%) to 91/105 (86.7%)), rhythm (50/90 (55.6%) to 85/105 (81.0%)), and rate (70/90 (77.8%) to 99/105 (94.3%)) (all p<0.01). Documentation of the PR interval, QRS complex, and axis also improved significantly. The proportion of patients diagnosed with ACS, including unstable angina, NSTEMI (non-ST-elevation myocardial infarction), and STEMI, decreased from 33/90 (36.7%) in the first cycle to 20/105 (19.0%) in the second cycle (p=0.007), while normal ECG findings increased, although not statistically significant.

Conclusion

Targeted quality improvement interventions resulted in significant enhancements in both the timeliness of ECG acquisition and the completeness of ECG documentation. These findings highlight the effectiveness of structured audit cycles and low-cost interventions in improving emergency cardiac care and emphasize the importance of sustained quality improvement efforts to ensure long-term impact.

## Introduction

Electrocardiography (ECG) is a fundamental diagnostic tool in the early evaluation of patients presenting with suspected acute coronary syndrome (ACS) in emergency departments (EDs). It provides rapid, non-invasive assessment of cardiac electrical activity and plays a pivotal role in identifying life-threatening conditions such as ST-elevation myocardial infarction (STEMI). Early diagnosis through ECG enables prompt initiation of reperfusion therapy, which is directly associated with improved clinical outcomes and reduced mortality [1,2].

International guidelines, including those from the American College of Cardiology (ACC) and the American Heart Association (AHA), recommend obtaining and interpreting a 12-lead ECG within 10 minutes of patient presentation to a healthcare facility [3,4]. Adherence to this time target is considered a key quality indicator in emergency cardiac care, as delays in ECG acquisition can lead to delayed diagnosis and treatment, ultimately increasing the risk of adverse outcomes [5].

Despite these well-established recommendations, multiple studies have demonstrated that compliance with the 10-minute ECG target remains suboptimal in many emergency settings [6]. Evidence suggests that a significant proportion of patients presenting with ischemic symptoms do not receive an ECG within the recommended timeframe, with reported delays extending far beyond guideline standards. Such delays may be attributed to factors including overcrowding, workflow inefficiencies, inadequate staffing, and lack of standardized protocols [5].

In addition to timeliness, the quality of ECG interpretation and documentation is equally critical. Accurate interpretation ensures appropriate clinical decision-making, while comprehensive documentation supports continuity of care, facilitates communication among healthcare providers, and provides medico-legal protection. Incomplete or inconsistent documentation of key ECG parameters, such as heart rate, rhythm, axis, and ST-segment changes, can compromise patient safety and hinder effective management.

Quality improvement (QI) initiatives have been widely recognized as effective strategies for addressing gaps in clinical practice. In particular, clinical audits structured around the Plan-Do-Study-Act (PDSA) framework enable systematic evaluation of current performance, implementation of targeted interventions, and reassessment to determine the impact of change [7]. Such closed-loop audit processes are essential for achieving sustainable improvements in healthcare delivery.

Previous quality improvement studies have demonstrated that interventions such as staff education, workflow optimization, and the introduction of standardized protocols can significantly reduce door-to-ECG times and improve adherence to guideline-recommended practices [5,8]. However, there remains a paucity of data from low-resource settings, where healthcare systems face additional challenges, such as limited staffing, high patient volume, constrained access to ECG machines, and lack of standardized triage protocols, that may impact the delivery of timely and high-quality care.

At baseline, the workflow for patients presenting to the ED was not supported by a standardized protocol for rapid ECG acquisition. Triage assessment was primarily based on initial clinical evaluation without a structured pathway to prioritize patients with suspected ACS for immediate ECG. ECG requests were typically initiated by the attending clinician after initial assessment, which could introduce delays, particularly during periods of high patient volume. In addition, ECG interpretation and documentation were performed without a standardized format, leading to variability in the completeness of recorded findings. These workflow limitations contributed to delays in ECG acquisition and inconsistencies in documentation, highlighting the need for a structured quality improvement approach.

Therefore, this study aimed to evaluate and improve the timeliness and quality of ECG acquisition, interpretation, and documentation in the ED of Hasahesa Teaching Hospital, Sudan. By conducting a closed-loop clinical audit within a quality improvement framework, this study sought to identify existing gaps, implement targeted interventions, and assess their effectiveness in aligning clinical practice with established international standards.

## Materials and methods

Study design and setting

This study was conducted as a closed-loop quality improvement project using a two-cycle clinical audit design structured around the PDSA framework [7]. The project was undertaken in the ED of Hasahesa Teaching Hospital, Sudan, a secondary-level healthcare facility that provides acute medical services to a high volume of patients, including those presenting with suspected ACS.

Study population and sampling strategy

The study population comprised all patients presenting to the ED during the defined audit periods who underwent electrocardiogram (ECG) assessment as part of their clinical evaluation, reflecting a broad range of clinical indications beyond suspected ACS. A total population sampling approach was employed, whereby all eligible cases within the study timeframe were included. This approach was selected to minimize selection bias and provide a comprehensive evaluation of real-world clinical practice. The study population predominantly consisted of adult patients, as pediatric ECG utilization in this setting was limited and not analyzed separately.

The first audit cycle was conducted over a three-month period from June to August 2025 and included 90 patients. Following this, a structured intervention phase was implemented over two months from September to October 2025. The second audit cycle was conducted over a subsequent three-month period from November 2025 to January 2026 and included 105 patients.

Audit standards and benchmark criteria

Audit standards were derived from internationally recognized clinical guidelines, particularly the American College of Cardiology/American Heart Association (ACC/AHA) recommendations for the management of ACS. These guidelines recommend that a 12-lead ECG should be obtained and interpreted within 10 minutes of patient arrival in patients with suspected ACS [4,9].

Accordingly, the primary benchmark for process efficiency in this study was the proportion of patients receiving ECG acquisition within 10 minutes of arrival. Additional standards included prompt interpretation following acquisition and immediate documentation of findings, reflecting best practice recommendations for timely clinical decision-making in emergency cardiac care [4,9].

Data collection procedures

Data were collected prospectively using a structured paper-based data collection form developed by the audit team for this study, capturing predefined parameters from patient records and ECG documentation during routine clinical care.

Data collection was conducted prospectively in real time during routine clinical practice throughout both audit cycles using paper-based clinical records, triage documentation, and ECG reports, as electronic patient records were not available in the study setting. Data were collected by members of the audit team independently from the clinicians responsible for ECG interpretation and documentation. Collection was performed throughout routine ED activity during the audit periods.

The first audit cycle was conducted over a three-month period, from June to August 2025, and included 90 patient cases. Following this, a two-month intervention and implementation phase was conducted from September to October 2025, during which educational sessions, workflow reinforcement, and documentation standardization strategies were introduced. The second audit cycle was subsequently conducted over a three-month period from November 2025 to January 2026 and included 105 patient cases. A total of 195 patient encounters were included across both cycles using a total population sampling approach.

Key process parameters were recorded at the point of care, including time from patient arrival to ECG acquisition, time from ECG acquisition to interpretation, and time from interpretation to documentation. These time intervals were determined using contemporaneously recorded timestamps from triage records, ECG printouts, and clinical notes, thereby minimizing recall bias and improving data accuracy. Although data regarding time from ECG acquisition to interpretation and from interpretation to documentation were collected as part of the audit process, these variables were not consistently documented in a standardized manner across both audit cycles and were therefore not included in the comparative results analysis.

Additional variables included the clinical indication for ECG, categorized as typical presentations (such as chest pain suggestive of ACS) or atypical presentations (including symptoms such as dyspnea, palpitations, dizziness, or epigastric discomfort), as well as the quality of ECG documentation. These variables were collected to characterize ECG utilization patterns within the ED, although subgroup analysis based on presentation type was beyond the scope of the current study. Documentation quality was assessed based on the completeness of essential ECG components, including heart rate, rhythm, electrical axis, ST-segment changes, QRS complex, and PR interval [9]. ACS was defined to include STEMI, non-STEMI (NSTEMI), and unstable angina. Classification was based on the final documented clinical diagnosis, incorporating ECG findings, clinical assessment, and, where available, cardiac biomarker results such as troponin levels.

To ensure consistency and reliability, data were collected using predefined criteria and standardized definitions for each parameter. Regular cross-checking of recorded data was performed where necessary to ensure completeness and accuracy.

Intervention

Following the analysis of baseline findings from the first audit cycle, a targeted multifaceted intervention was implemented over a two-month period from September to October 2025 to address deficiencies identified in ECG workflow, timeliness, and documentation practices. Baseline audit findings demonstrated delays in ECG acquisition, inconsistent documentation of key ECG parameters, and the absence of standardized workflow reinforcement within the ED. Informal discussions with ED staff further identified contributing factors, including limited awareness of recommended ECG timing targets, variability in documentation practices, and lack of structured prioritization for patients requiring urgent ECG assessment.

The intervention consisted primarily of structured educational sessions delivered to doctors and nurses working within the ED, as these staff members were directly involved in ECG acquisition, interpretation, and documentation. Sessions were conducted in small groups during routine departmental activities to accommodate varying clinical schedules and workflow demands. Teaching focused on the importance of timely ECG acquisition in accordance with international guidelines, particularly the recommended 10-minute target for suspected ACS, as well as standardized ECG interpretation and documentation practices. Specific emphasis was placed on recognition and documentation of ST-segment elevation and other clinically significant ECG abnormalities.

In addition, visual reminder materials and workflow prompts were displayed in key clinical areas within the ED, including triage and ECG assessment areas, to reinforce awareness of recommended ECG timing targets and documentation standards (Appendix 1). Standardized documentation reinforcement focused on ensuring inclusion of essential ECG interpretation components, including heart rate, rhythm, axis, PR interval, QRS complex, and ST-segment findings.

Audit feedback was delivered collectively during departmental discussions and educational meetings, highlighting deficiencies identified during the first audit cycle and reinforcing adherence to recommended practices. These interventions were selected as practical, low-cost strategies appropriate for the resource limitations of the study setting and were implemented in accordance with established quality improvement principles to improve workflow efficiency and adherence to clinical standards.

Re-audit (second cycle)

The second audit cycle was conducted over a three-month period following completion of the intervention phase and included 105 patient cases. The same methodology, data collection tools, audit parameters, and inclusion criteria used in the first cycle were applied to ensure direct comparability between cycles.

This re-audit allowed for objective evaluation of the effectiveness of the implemented interventions in improving the timeliness of ECG acquisition, interpretation, and documentation, as well as the completeness of ECG reporting.

Data analysis

Data were analyzed using descriptive and comparative statistical methods. Continuous variables, including time intervals, were summarized using appropriate measures of central tendency. Categorical variables were expressed as frequencies and percentages.

Comparisons between the first and second audit cycles were performed to assess changes in performance across all measured parameters. The chi-square (χ²) test was used to evaluate differences in categorical variables between the two cycles. Where expected cell counts were less than 5, Fisher’s exact test was applied as appropriate.

A p-value of less than 0.05 was considered statistically significant. The results were interpreted in the context of both statistical significance and clinical relevance to assess the impact of the quality improvement interventions.

Ethical considerations

This study was conducted as a quality improvement audit utilizing anonymized data derived from routine clinical records, with no direct patient interaction or intervention. Ethical approval was obtained from the Institutional Review Board (IRB) of Hasahesa Teaching Hospital, Gezira State, Sudan (Ref. No.: HTH/IRB/2025/037).

The study adhered to the ethical principles outlined in the Declaration of Helsinki. As the project was classified as a quality improvement initiative, the requirement for individual informed consent was waived. All data were anonymized prior to analysis to ensure patient confidentiality.

## Results

A total of 195 patient encounters were analyzed across two audit cycles, including 90 cases in the first cycle and 105 cases in the second cycle.

Significant improvements were observed in the timeliness of ECG acquisition following the implementation of targeted interventions. The proportion of patients who underwent ECG within 10 minutes of arrival increased from 43/90 (47.8%) in the first cycle to 90/105 (85.7%) in the second cycle, representing an absolute improvement of 37.9% (χ² = 28.6, df = 1, p < 0.001). In parallel, the mean time from arrival to ECG acquisition decreased from 25.4 minutes to 4.5 minutes.

Notable enhancements were also demonstrated in the quality of ECG documentation. Documentation of cardiac rhythm improved from 50/90 (55.6%) to 85/105 (81.0%) (χ² = 13.9, df = 1, p < 0.001), while documentation of heart rate increased from 70/90 (77.8%) to 99/105 (94.3%) (χ² = 9.5, df = 1, p = 0.002). The most pronounced improvement was observed in ST-segment documentation, which increased from 25/90 (27.8%) to 91/105 (86.7%) (χ² = 64.2, df = 1, p < 0.001).

Similarly, significant improvements were observed in other ECG parameters. Documentation of the PR interval increased from 10/90 (11.1%) to 50/105 (47.6%) (χ² = 28.1, df = 1, p < 0.001), QRS complex documentation improved from 16/90 (17.8%) to 60/105 (57.1%) (χ² = 31.4, df = 1, p < 0.001), and axis documentation increased from 20/90 (22.2%) to 50/105 (47.6%) (χ² = 13.5, df = 1, p < 0.001).

With respect to diagnostic outcomes, the proportion of patients diagnosed with ACS, including ST-elevation myocardial infarction (STEMI), non-ST-elevation myocardial infarction (NSTEMI), and unstable angina, decreased from 33/90 (36.7%) in the first cycle to 20/105 (19.0%) in the second cycle (χ² = 7.2, df = 1, p = 0.007). The proportion of normal ECG findings increased from 10/90 (11.1%) to 22/105 (21.0%), although this change did not reach statistical significance (χ² = 3.4, df = 1, p = 0.06). ECG findings not classified as ACS-related diagnoses showed a slight reduction from 57/90 (63.3%) in the first cycle to 58/105 (55.2%) in the second cycle, which was not statistically significant (χ² = 1.3, df = 1, p = 0.25).

Overall, these findings demonstrate a substantial improvement in both the efficiency of ECG acquisition and the completeness of ECG documentation following the implementation of targeted quality improvement interventions (Table 1).

**Table 1:** Comparison of ECG Timeliness, Documentation Quality, and Diagnostic Outcomes Between Audit Cycles Before and After Quality Improvement Intervention Comparison of key performance indicators related to electrocardiogram (ECG) acquisition, interpretation, and documentation between the first and second audit cycles. The table presents frequencies and percentages for categorical variables, along with absolute percentage changes. Statistical comparisons between cycles were performed using the chi-square test, with p-values indicating the significance of observed differences. ACS: acute coronary syndrome; ECG: electrocardiogram

Parameter		First Cycle (n=90)	Second Cycle (n=105)	Change (%)	Interpretation	χ² (df = 1)	p-value
Time from arrival to ECG acquisition	Time from arrival to ECG ≤10 min	43 (47.8%)	90 (85.7%)	+37.9%	Marked improvement in timely ECG acquisition	28.6	<0.001
	Average time (minutes)	25.4	4.5	-	Significant reduction in delay	-	<0.001
Documentation quality	Rhythm documented	50 (55.6%)	85 (81.0%)	+25.4%	Improved documentation completeness	13.9	<0.001
	ST-segment documented	25 (27.8%)	91 (86.7%)	+58.9%	Substantial improvement	64.2	<0.001
	PR interval documented	10 (11.1%)	50 (47.6%)	+36.5%	Significant improvement	28.1	<0.001
	PR interval documented	10 (11.1%)	50 (47.6%)	+36.5%	Significant improvement	28.1	<0.001
	QRS complex documented	16 (17.8%)	60 (57.1%)	+39.3%	Marked improvement	31.4	<0.001
	Axis documented	20 (22.2%)	50 (47.6%)	+25.4%	Moderate improvement	13.5	<0.001
Diagnostic findings	Normal ECG	10 (11.1%)	22 (21.0%)	+9.9%	Non-significant increase	3.4	0.06
	ACS	33 (36.7%)	20 (19.0%)	-17.7%	Reduction in ACS proportion	7.2	0.007
	Other causes	57 (63.3%)	58 (55.2%)	-8.1%	Non-significant increase	1.3	0.25

## Discussion

This study demonstrates a substantial improvement in both the timeliness of ECG acquisition and the quality of ECG documentation following the implementation of targeted interventions, including staff education, workflow reinforcement, audit feedback, and standardized ECG documentation practices, in a Sudanese ED. These findings reinforce the critical role of structured audit cycles and educational interventions in addressing gaps in acute cardiac care, particularly in resource-limited settings.

At baseline, the proportion of patients receiving ECG within 10 minutes of arrival was 47.8%, with a mean time of 25.4 minutes, reflecting significant delays in initial cardiac assessment. These findings are consistent with previous literature demonstrating suboptimal adherence to guideline-recommended door-to-ECG times. For example, Diercks et al. reported that only approximately one-third of patients presenting with chest pain received an ECG within 10 minutes of arrival, highlighting a widespread challenge in emergency care systems [10].

Following the implementation of targeted interventions, our study demonstrated a marked improvement, with 85.7% of patients achieving ECG acquisition within 10 minutes and a reduction in mean time to 4.5 minutes. This magnitude of improvement is consistent with findings from Idrees et al. (2025), who reported an increase from 4% to 81.8% compliance with the 10-minute ECG target after introducing similar interventions, including educational sessions and workflow optimization [11].

Our findings support existing evidence that structured interventions, particularly those focused on reinforcement of ECG prioritization practices, staff awareness, and triage-based workflow reinforcement, can significantly reduce delays in ECG acquisition. A systematic review by Chhabra et al. further demonstrated that interventions such as triage education, dedicated ECG personnel, and protocol standardization are highly effective in improving door-to-ECG times in emergency settings [12]. In our study, the intervention primarily incorporated triage-focused education, reinforcement of ECG prioritization practices, and increased awareness of recommended ECG timing standards, while dedicated ECG personnel and formal protocol restructuring were not feasible because of resource and staffing limitations [12].

In addition to improvements in timeliness, our study revealed significant enhancement in the quality of ECG documentation across all assessed parameters. The most notable improvement was observed in ST-segment documentation, which increased from 27.8% to 86.7%. Improvements were also observed in rhythm, rate, PR interval, QRS complex, and axis documentation, although these were less pronounced than the improvement observed in ST-segment documentation. This may reflect the greater clinical emphasis placed on the recognition and documentation of ST-segment abnormalities during the educational intervention, given their immediate relevance to ACS assessment and urgent management decisions. These findings are broadly comparable to those reported by Idrees et al., where documentation completeness improved markedly following structured educational interventions, with several documentation parameters approaching near-complete compliance [11]. However, complete compliance was not consistently achieved in our study, possibly due to higher patient volume, resource limitations, variable staff workload, and operational pressures in the ED.

The improvement in documentation quality can be attributed to increased awareness and reinforcement of standardized ECG interpretation practices. Previous studies have highlighted that inadequate ECG interpretation and documentation are often linked to insufficient training and a lack of structured protocols and may contribute to delayed recognition of clinically significant findings, impaired continuity of care, and communication errors between healthcare providers. Educational interventions targeting these areas have been shown to significantly improve both clinician competency and documentation practices [13].

Furthermore, quality improvement initiatives conducted in other settings have demonstrated similar benefits. A multicenter study in Saudi Arabia reported that system-level interventions, including workflow redesign, improved interdepartmental coordination, and increased resource availability, significantly increased the proportion of patients receiving timely ECGs [14]. Similarly, studies have shown that strategies such as optimized triage pathways, improved access to ECG machines, and staff education can substantially reduce delays in ECG acquisition [15].

Despite these improvements, it is important to recognize that achieving optimal ECG performance, defined as timely ECG acquisition, accurate interpretation, and complete documentation in accordance with recommended clinical standards, remains a complex, multifactorial challenge. Previous research by Zègre-Hemsey et al. demonstrated that only 59% of patients with ischemic symptoms received an ECG within the recommended timeframe, with disparities observed based on gender and presentation characteristics [6]. These findings highlight the need for continuous monitoring and targeted interventions to ensure equitable and consistent care delivery. Although subgroup analysis based on typical and atypical clinical presentations was beyond the scope of the current study, future research exploring disparities in ECG acquisition and documentation across different presentation patterns may provide important additional insights for targeted quality improvement initiatives.

Interestingly, our study also demonstrated a reduction in the proportion of patients diagnosed with ACS in the second cycle. This finding should be interpreted cautiously and may partly reflect the inclusion of all patients undergoing ECG assessment rather than only those presenting with chest pain, as well as the increased emphasis on recognizing and documenting ST-segment abnormalities during the intervention phase. Seasonal variation between audit periods cannot be completely excluded, although the study was not specifically designed to assess seasonal trends. In addition, while no major staffing changes occurred between audit cycles, routine variation in ED personnel may have influenced workflow and documentation practices. Similar observations were noted in previous audits, where improved diagnostic processes led to changes in case distribution, including increased identification of normal ECG findings [11].

Overall, the findings of this study demonstrate improved adherence to international guideline-recommended practices following targeted educational interventions, workflow reinforcement, and standardized ECG documentation strategies in the ED setting. The ACC/AHA guidelines highlight that adherence to the 10-minute ECG target is critical for early diagnosis and timely intervention, ultimately improving patient outcomes [4,9].

Limitation

This study has several limitations that should be considered when interpreting the findings. Although the audit demonstrated significant improvements following intervention, it was conducted in a single center, which may limit the generalizability of the results to other healthcare settings. The relatively moderate sample size may also reduce the external validity of the findings.

Additionally, while prospective real-time data collection improved the completeness and consistency of recorded audit data, the absence of randomization and blinding introduces the potential for observer and documentation bias. The possibility of a Hawthorne effect, whereby healthcare providers modify their behavior due to awareness of being observed, cannot be excluded.

The study focused primarily on process measures, such as time intervals and documentation quality, without assessing downstream clinical outcomes such as door-to-balloon time, morbidity, or mortality. Furthermore, potential confounding factors, including patient case-mix, staffing levels, and workload variations, were not controlled for.

Finally, the sustainability of the observed improvements over the long term was not evaluated, highlighting the need for ongoing monitoring and repeated audit cycles to ensure that gains in performance are maintained.

## Conclusions

This closed-loop clinical audit demonstrated that targeted interventions, including staff education, reinforcement of ECG prioritization practices, visual reminder materials, audit feedback, and standardized ECG documentation reinforcement, were associated with substantial improvements in both the timeliness of ECG acquisition and the quality of ECG documentation in the ED setting. The marked increase in compliance with the recommended 10-minute ECG target, alongside significant improvements in documentation completeness, highlights the potential impact of structured audit-based interventions in improving adherence to recommended clinical standards.

These findings underscore the critical importance of implementing protocol-driven approaches and sustained quality improvement initiatives to enhance adherence to clinical guidelines, particularly in resource-limited settings. The study highlights that relatively simple, low-cost interventions can produce meaningful and measurable improvements in the early assessment of patients with suspected ACS. Ongoing monitoring, reinforcement of best practices, and repeated audit cycles are essential to ensure the sustainability of these improvements and to further advance the quality of emergency cardiac care.

## Figures and Tables

**Figure 1:**
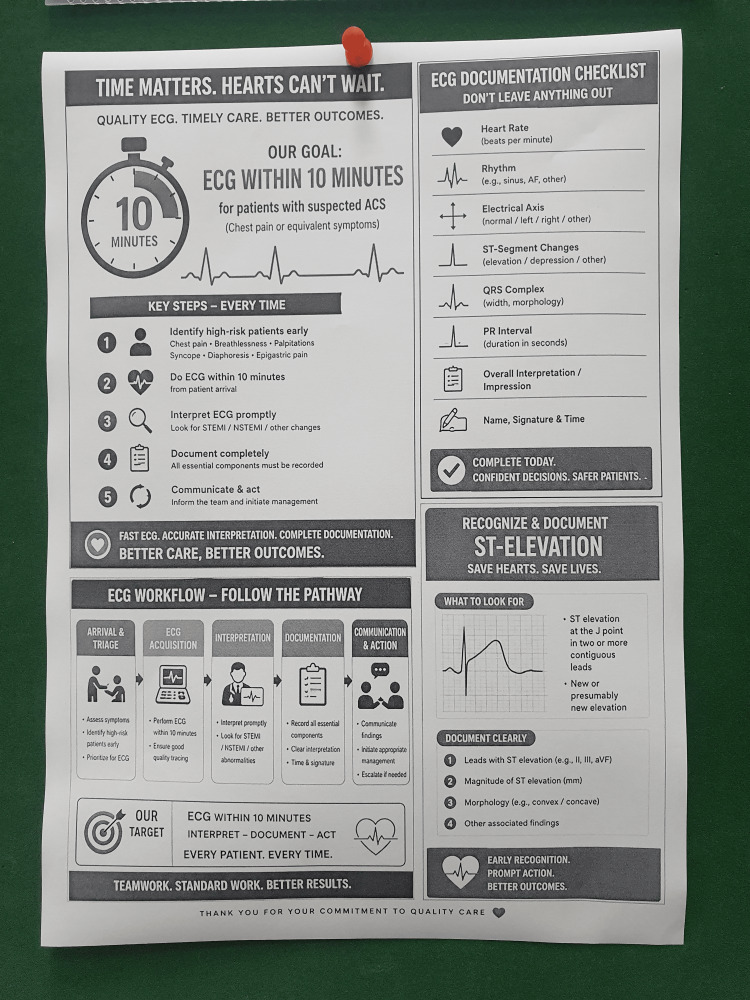
An example of the educational reminder poster used during the intervention phase. Actual photograph taken in the emergency department at Hasahesa Teaching Hospital during the intervention phase, which shows the actual educational reminder materials and workflow prompt.

## Appendices

Appendix 1
